# Supplementary material for: Glycine Cleavage System and cAMP Receptor Protein Co-Regulate CRISPR/cas3 Expression to Resist Bacteriophage
Source: Viruses. 2020 Jan 13;12(1):90. doi: 10.3390/v12010090 (PMC7019758; doi:10.3390/v12010090)
Supplement: Supplementary file 1 [file viruses-12-00090-s001.zip › Fig. S2.docx]

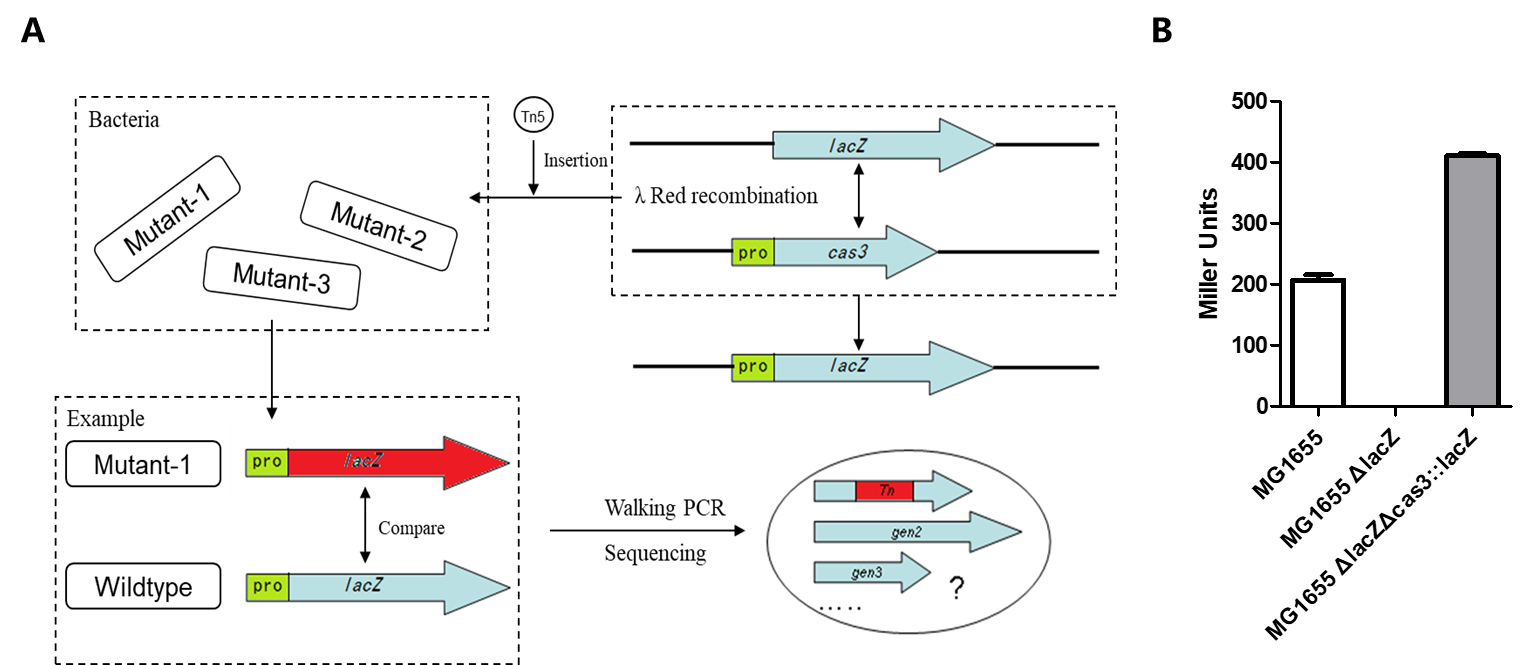


**Supplementary Figure 2.** Construction of E. coli MG1655Δ*lacZ*Δ*cas3*::*lacZ* reporter bacterium. **(A)** Schematic diagram of MG1655Δ*lacZ*Δ*cas3*::*lacZ* reporter bacterium construction. **(B)** The β-gal activities of WT and mutants were measured. The mutation of *lacZ* led to loss of β-galactosidase activity. Replacing *cas3* with *lacZ* restored β-galactosidase activity. All the data were mean±SEM of at least three replicates, and P value (*p*＜0.05) was analysed by t-test.
