# Supplementary material for: Glycine Cleavage System and cAMP Receptor Protein Co-Regulate CRISPR/cas3 Expression to Resist Bacteriophage
Source: Viruses. 2020 Jan 13;12(1):90. doi: 10.3390/v12010090 (PMC7019758; doi:10.3390/v12010090)
Supplement: Supplementary file 1 [file viruses-12-00090-s001.zip › Fig. S3.docx]

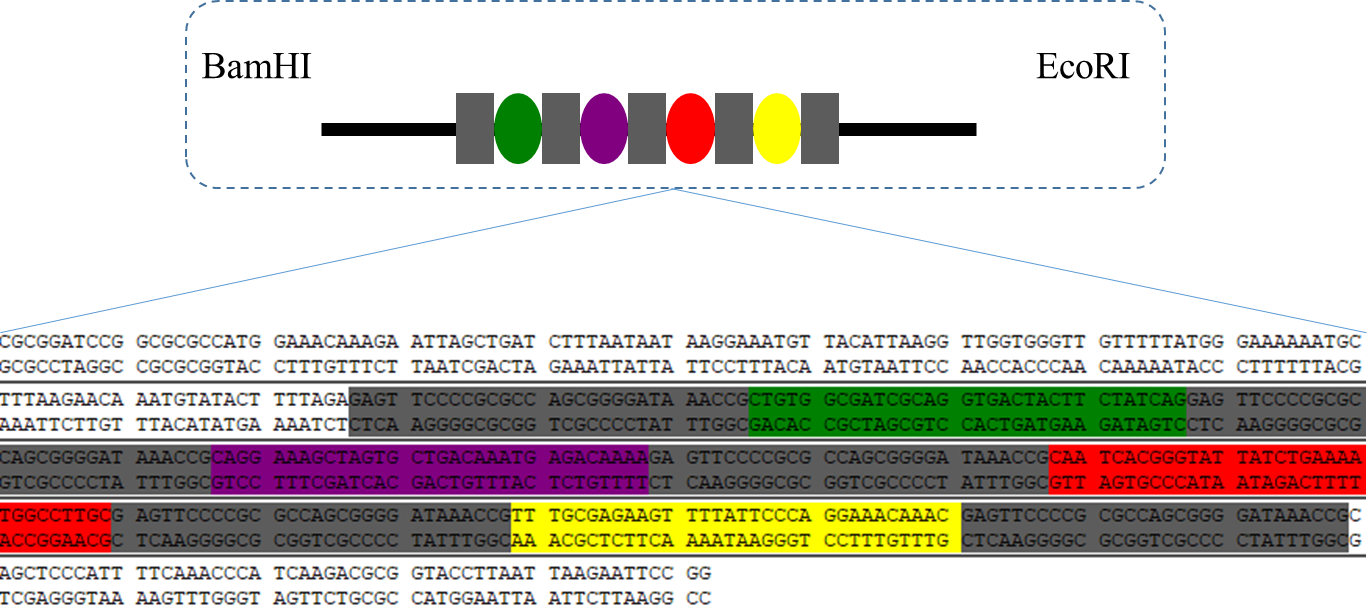


**Supplementary Figure 3.** Schematic diagram of anti-vB_EcoS_SH2 spacer plasmid construction. Grey area represented repeated sequences. Green area represented ORF28 (phage minor capsid protein). Purple area represented ORF48. Red area represented ORF50 (phage exonuclease). Yellow area represented ORF58 (DNA adenine methyltransferase, phage-associated).
