# Supplementary material for: Glycine Cleavage System and cAMP Receptor Protein Co-Regulate CRISPR/cas3 Expression to Resist Bacteriophage
Source: Viruses. 2020 Jan 13;12(1):90. doi: 10.3390/v12010090 (PMC7019758; doi:10.3390/v12010090)
Supplement: Supplementary file 1 [file viruses-12-00090-s001.zip › Fig. S4.docx]

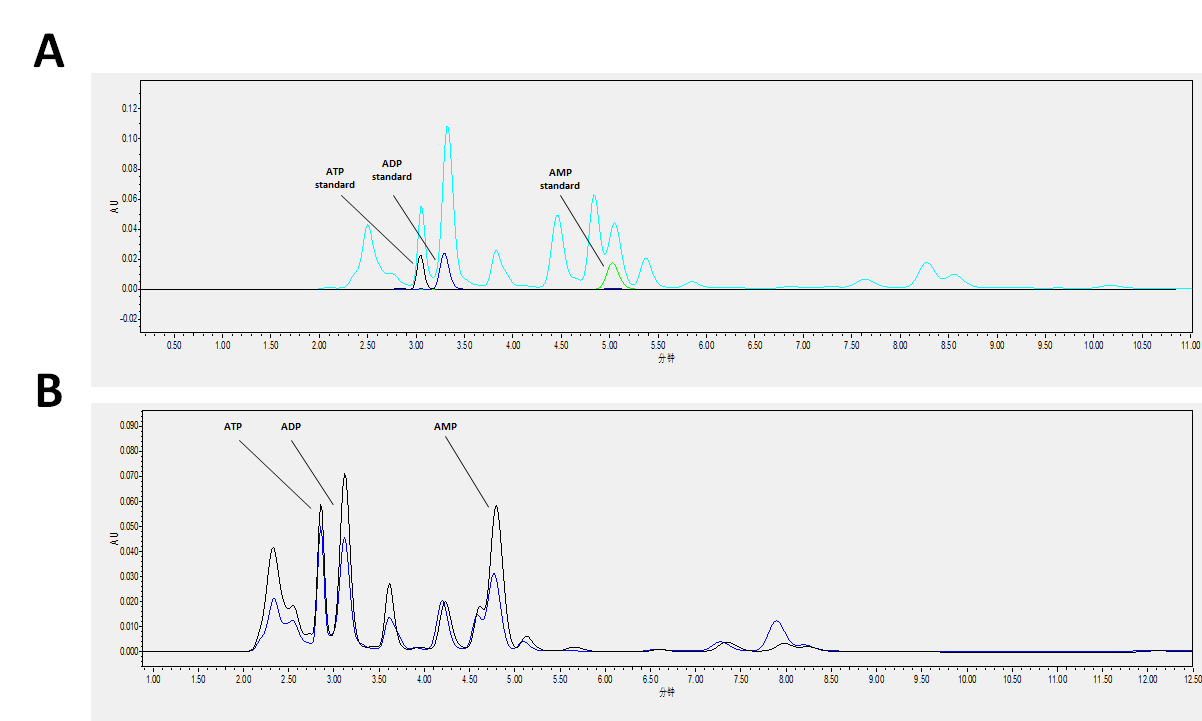


**Supplementary Figure 4.** Determination of ATP, ADP and AMP in WT and Δ*gcvP* by High Performance Liquid Chromatography. **(A)** Determination of the elution time of the standards **(B)** Determination of the elution time of ATP, ADP and AMP in WT and Δ*gcvP*. The black peaks represent the WT and the blue peaks represent the Δ*gcvP*. All the data were mean ± SEM of at least three replicates, and P value (*p*＜0.05) was analysed by t-test.
