# Supplementary material for: Glycine Cleavage System and cAMP Receptor Protein Co-Regulate CRISPR/cas3 Expression to Resist Bacteriophage
Source: Viruses. 2020 Jan 13;12(1):90. doi: 10.3390/v12010090 (PMC7019758; doi:10.3390/v12010090)
Supplement: Supplementary file 1 [file viruses-12-00090-s001.zip › Supplementary Table S1.docx]

**Supplementary Table S1**. Strains used in this study.

| **Strain** | **Genotype/Phenotype** | **Reference** |
| --- | --- | --- |
| MG1655 | *F-, λ-, ilvG-, rfb-50, rph-1* | ([1](#_ENREF_1)) |
| MC1061 | *hsdR2*, *hsdM+*, *hsdS+*, *araD139*, Δ*(ara-leu)7697*, Δ*(lac)X74*, *galE15*, *galK16*, *rpsL*, *(StrR)*, *mcrA*,  *mcrB1* | ([1](#_ENREF_1)) |
| DH5α | F^-^, φ80Δd*lacZ*M15, Δ(*lacZYA*–*argF*)U169, *endA1*, *recA1*, *hsd*R17 (r_K_^-^m_K_^+^), *deoR*, *thi-1*, *sup*E44, λ^-^, *gyr*A96, *rel*A1 | Transgen |
| BL21 | *F*-, *ompT*, *hsdS*(*rB*-, *mB*-), *gal*, *dcm*(DE3) | Transgen |
| S17-1 λ*pir* | *recA, pro,hsdR, recA::*RP4-2-Tc::Mu, λ*pir,* Tmp^R^, Sp^R^, Sm^R^ | ([2](#_ENREF_2)) |
| **MG1655 derivatives** | | |
| EC1001  EC1002 | Δ*lacZ*  Δ*lacZ*Δ*cas3*::*lacZ*, used for transposon mutagenesis | This study  ([3](#_ENREF_3)) |
| EC1003 | Δ*lacZ*Δ*cas3*::*lacZ, gcvT*::Tn5-Cm, Cm^R^, Nal^R^ | This study |
| EC12 | Δ*lacZ,* pRCL2, Cm^R^ | This study |
| EC17  EC18 | Δ*lacZ,* pRCL7, Cm^R^  Δ*lacZ,* pRCL8, Cm^R^ | This study  This study |
| EC20 | ΔlacZ,Δ*gcvP* | This study |
| EC30 | Δ*lacZ*,Δ*gcvP*, pBAD-*gcvP*, Amp^R^ | This study |
| EC40 | Δ*lacZ*,Δ*gcvT* | This study |
| EC50 | Δ*lacZ*,Δ*gcvP*, pBAD-*gcvT*, Amp^R^ | This study |
| EC60 | Δ*lacZ*Δ*crp* | This study |
| EC70 | Δ*lacZ*,Δ*crp*, pBAD-*crp*, Amp^R^ | This study |
| EC80 | Δ*lacZ*Δ*crp*Δ*gcvP* | This study |
| EC90 | Δ*lacZ*Δ*hns* | This study |
| EC100 | Δ*lacZ*Δ*hns*Δ*gcvP* | This study |
| EC110 | Δ*lacZ*Δ*hns*Δ*crp* | This study |
| EC120 | Δ*lacZ*Δ*hns*Δ*cas3* | This study |
| EC130 | Δ*lacZ*Δ*cas3* | This study |
| EC150 | Δ*lacZ*Δ*crp*Δ*gcvP*, pBAD-*gcvP*, Amp^R^ | This study |
| EC151 | Δ*lacZ*Δ*crp*Δ*gcvP*, pBAD-*gcvP*, pRCL1, Amp^R^, Cm^R^ | This study |
| EC11 | Δ*lacZ*, pRCL1, Cm^R^ | This study |
| EC21 | Δ*lacZ*Δ*gcvP*, pRCL1, Cm^R^ | This study |
| EC31 | Δ*lacZ*Δ*gcvP*, pBAD-*gcvP*, pRCL1, Amp^R^, Cm^R^ | This study |
| EC41 | Δ*lacZ*Δ*gcvT*, pRCL1, Cm^R^ | This study |
| EC51 | Δ*lacZ*Δ*gcvP*, pBAD-*gcvT*, pRCL1, Amp^R^, Cm^R^ | This study |
| EC61 | Δ*lacZ*Δ*crp*, pRCL1, Cm^R^ | This study |
| EC71 | Δ*lacZ*Δ*crp*, pBAD-*crp*, pRCL1, Amp^R^, Cm^R^ | This study |
| EC19 | Δ*lacZ*, pRCL9, Cm^R^ | This study |
| EC69 | Δ*lacZ*Δ*crp*, pRCL9, Cm^R^ | This study |
| EC81 | Δ*lacZ*Δ*crp*Δ*gcvP*, pRCL1, Cm^R^ | This study |
| EC91 | Δ*lacZ*Δ*hns*, pRCL1, Cm^R^ | This study |
| EC101 | Δ*lacZ*Δ*hns*Δ*gcvP*, pRCL1, Cm^R^ | This study |
| EC111 | Δ*lacZ*Δ*hns*Δ*crp*, pRCL1, Cm^R^ | This study |
| EC13 | Δ*lacZ*, pRCL3, Cm^R^ | This study |
| EC23 | Δ*lacZ*Δ*gcvP*, pRCL3, Cm^R^ | This study |
| EC14 | Δ*lacZ*, pRCL4, Cm^R^ | This study |
| EC24 | Δ*lacZ*Δ*gcvP*, pRCL4, Cm^R^ | This study |
| EC64 | Δ*lacZ*Δ*crp*, pRCL4, Cm^R^ | This study |
| EC94 | Δ*lacZ*Δ*hns*, pRCL4, Cm^R^ | This study |
| EC104 | Δ*lacZ*Δ*hns*Δ*gcvP*, pRCL4, Cm^R^ | This study |
| EC114 | Δ*lacZ*Δ*hns*Δ*crp*, pRCL4, Cm^R^ | This study |
| EC15 | Δ*lacZ*, pGEX, Amp^R^ | This study |
| EC25 | Δ*lacZ*Δ*gcvP*, pGEX, Amp^R^ | This study |
| EC65 | Δ*lacZ*Δ*crp*, pGEX, Amp^R^ | This study |
| EC135 | Δ*lacZ*Δ*cas3*, pGEX, Amp^R^ | This study |
| EC95 | Δ*lacZ*Δ*hns*, pGEX, Amp^R^ | This study |
| EC105 | Δ*lacZ*Δ*hns*Δ*gcvP*, pGEX, Amp^R^ | This study |
| EC115 | Δ*lacZ*Δ*hns*Δ*crp*, pGEX, Amp^R^ | This study |
| EC125 | Δ*lacZ*Δ*hns*Δ*cas3*, pGEX, Amp^R^ | This study |
| EC16 | Δ*lacZ*, pGEX3, Amp^R^ | This study |
| EC26 | Δ*lacZ*Δ*gcvP*, pGEX3, Amp^R^ | This study |
| EC66 | Δ*lacZ*Δ*crp*, pGEX3, Amp^R^ | This study |
| EC136 | Δ*lacZ*Δ*cas3*, pGEX3, Amp^R^ | This study |
| EC96 | Δ*lacZ*Δ*hns*, pGEX3, Amp^R^ | This study |
| EC106 | Δ*lacZ*Δ*hns*Δ*gcvP*, pGEX3, Amp^R^ | This study |
| EC116 | Δ*lacZ*Δ*hns*Δ*crp*, pGEX3, Amp^R^ | This study |
| EC126 | Δ*lacZ*Δ*hns*Δ*cas3*, pGEX3, Amp^R^ | This study |
| EC141  EC142  EC143  EC144  EC145  EC146 | Δ*lacZ*, pBAD-*leuO*, pGEX, Amp^R^, Cm^R^  Δ*lacZ*Δ*gcvP*, pBAD-*leuO*, pGEX, Amp^R^, Cm^R^  Δ*lacZ*Δ*crp*, pBAD-*leuO*, pGEX, Amp^R^, Cm^R^  Δ*lacZ*, pBAD-*leuO*, pGEX3, Amp^R^, Cm^R^  Δ*lacZ*Δ*gcvP*, pGEX3, Amp^R^, Cm^R^  Δ*lacZ*Δ*crp*, pGEX3, Amp^R^, Cm^R^ | This study  This study  This study  This study  This study  This study |

**References**

1. **Fu Q, Li SY, Wang ZF, Shan WY, Ma JJ, Cheng YQ, Wang HG, Yan YX, Sun JH.** 2017. H-NS Mutation-Mediated CRISPR-Cas Activation inhibits Phage Release and Toxin Production of Escherichia coli Stx2 Phage Lysogen. Front Microbiol **8**.

2. **Simon R, Priefer U, Pühler A.** 1983. A Broad Host Range Mobilization System for In Vivo Genetic Engineering: Transposon Mutagenesis in Gram Negative Bacteria. Nature Biotechnology **1:**784-791.

3. **Patterson AG, Chang JT, Taylor C, Fineran PC.** 2015. Regulation of the Type I-F CRISPR-Cas system by CRP-cAMP and GalM controls spacer acquisition and interference. Nucleic Acids Res **43:**6038-6048.
