# Supplementary material for: Glycine Cleavage System and cAMP Receptor Protein Co-Regulate CRISPR/cas3 Expression to Resist Bacteriophage
Source: Viruses. 2020 Jan 13;12(1):90. doi: 10.3390/v12010090 (PMC7019758; doi:10.3390/v12010090)
Supplement: Supplementary file 1 [file viruses-12-00090-s001.zip › Supplementary Table S2.docx]

**Supplementary Table S2**. Plasmids used in this study.

| **Plasmid** | **Description** | **Reference** |
| --- | --- | --- |
| pKD46 | λ Red recombinase expression, Amp^R^ | ([1](#_ENREF_1)) |
| pKD3 | Template of the chloramphenicol resistance cassette, Cm^R^ | ([1](#_ENREF_1)) |
| pCP20 | FLP recombinase expression, Amp^R^, Cm^R^ | ([1](#_ENREF_1)) |
| pET28a | Expression vector, Kan^R^ | Novagen |
| pET28a-*cas3* | pET28a cloned with ORF of *cas3*, Kan^R^ | This study |
| pRCL | β-galactosidase reporter vector constructed by our laboratory. For detection of promoter activation, p15A origin, *rrnB* terminator, Cm^R^ | This study |
| pRCL1 | pRCL cloned with -169 to 0 of *cas3*, Cm^R^ | This study |
| pRCL7 | pRCL cloned with -229 to 0 of *cas3*, Cm^R^ | This study |
| pRCL8 | pRCL cloned with -439 to 0 of *cas3*, Cm^R^ | This study |
| pRCL2 | pRCL cloned with -511 to 0 of *gcvTHP*, Cm^R^ | This study |
| pRCL3 | pRCL cloned with -1270 to 0 of *crp*, Cm^R^ | This study |
| pRCL4 | pRCL cloned with -510 to 0 of *casA*, Cm^R^ | This study |
| pRCL9 | pRCL cloned with -169 to 0 of mutant *cas3*, Cm^R^ | This study |
| pGEX | Expression vector pGEX-6p-1, Amp^R^ | This study |
| pGEX3 | pGEX-6p-1 cloned with anti-vB_EcoS_SH2 spacer, Amp^R^ | This study |
| pGEX1 | pGEX-6p-1 cloned with CRIPSR1 loci of MG1655, Amp^R^ | This study |
| pGEX2 | pGEX-6p-1 cloned with CRIPSR2 loci of MG1655, Amp^R^ | This study |
| pBAD  pBAD(Cm) | Expression vector derived from pBAD24, Δ*araC*, Amp^R^  Expression vector derived from pBAD24, Δ*araC*, Cm^R^ | This study  This study |
| pBAD-*gcvP* | pBAD cloned with ORF and putative promoter of *gcvP*, Amp^R^ | This study |
| pBAD-*gcvT* | pBAD cloned with ORF and putative promoter of *gcvT*, Amp^R^ | This study |
| pBAD-*crp*  pBAD-*leuO* | pBAD cloned with ORF and putative promoter of *crp*, Amp^R^  pBAD(Cm) cloned with ORF and putative promoter of *leuO*, Amp^R^ | This study  This study |

**References**

1. **Datsenko KA, Wanner BL.** 2000. One-step inactivation of chromosomal genes in Escherichia coli K-12 using PCR products. P Natl Acad Sci USA **97:**6640-6645.
