# Supplementary material for: Glycine Cleavage System and cAMP Receptor Protein Co-Regulate CRISPR/cas3 Expression to Resist Bacteriophage
Source: Viruses. 2020 Jan 13;12(1):90. doi: 10.3390/v12010090 (PMC7019758; doi:10.3390/v12010090)
Supplement: Supplementary file 1 [file viruses-12-00090-s001.zip › Supplementary Table S3.docx]

**Supplementary Table S3**. Oligonucleotide primers used in this study.

| **Name** | **Sequence (5'-3') (**Restriction site(s) underlined) | **Notes** |
| --- | --- | --- |
| P01 | TATGTTGTGTGGAATTGTGAGCGGATAACAATTTCACACAGGAAACAGCT GTGTAGGCTGGAGCTGCTT | F^[[1]](#footnote-1)^ for Δ*lacZ* Cm cassette |
| P02 | ATGGATTTCCTTACGCGAAATACGGGCAGACATGGCCTGCCCGGTTATTACATATGAATATCCTCCTTAG | R^[[2]](#footnote-2)^ for Δ*lacZ* Cm cassette |
| P03 | ATGCCATTGCTGTGGAAGCT | F for detection of Δ*lacZ* |
| P04 | CTGAACAGTTCCAGTGCCAG | R for detection of Δ*lacZ* |
| P11 | CAAAGTCGAACCGATGAACC | F for *lacZ* Cm cassette |
| P12 | TGAATCCGTAATCATGGTCATTAATAGCCTCCCTGTTTTTT | R for *lacZ* Cm cassette |
| P13 | AAAAAACAGGGAGGCTATTAATGACCATGATTACGGATTCA | F for *lacZ* Cm cassette |
| P14 | GAAGCAGCTCCAGCCTACACTTATTTTTGACACCAGACCA | R for *lacZ* |
| P15 | TGGTCTGGTGTCAAAAATAAGTGTAGGCTGGAGCTGCTTC | F for Cm cassette |
| P16 | TGATAACAATCATTCCCGAAGCATATGAATATCCTCCTTAG | R for *lacZ* Cm cassette |
| P17 | CTAAGGAGGATATTCATATGCTTCGGGAATGATTGTTATCA | F for *lacZ* Cm cassette |
| P18 | CAGTGAGCGGATTCATTATG | R for *lacZ* Cm cassette |
| P19 | AAGCTCAACCTGAAAGTGTACCG | F for detection of Δ*lacZ*Δ*cas3*::*lacZ* |
| P110 | TTGACACCTTTGGTCTGCAT | R for detection of Δ*lacZ*Δ*cas3*::*lacZ* |
| P21 | ATGCTGGCAATCACTTTGACGTG | Primer of Tn5 for walking PCR |
| P22 | CTTGAGGCGTTGCATCCAGGTC | Primer of Tn5 for walking PCR |
| P23 | CACCGAACAAGTGATCCCGATGT | Primer of Tn5 for walking PCR |
| P31 | ACTAAGCTTTGATGGTTTGGTCTCCTGTA | F for -169 to 0 of *cas3* cloning to pRCL(HindIII) |
| P32 | ACTAAGCTTGTTGAGTATCAGAGAGCGTG | F for -229 to 0 of c*as3* cloning to pRCL(HindIII) |
| P33 | ACTAAGCTTCTATCAACCTGAGGCTCGC | F for -439 to 0 of *cas3* cloning to pRCL(HindIII) |
| P34  P35  P36 | CAGGGATCCATTTTAAAAATTATCTGTG  GCCTCTTCGCTATTACGCCA  TGTGCTTCTCAAATGCCTG | R for putative promoter of *cas3* cloning to pRCL(BamHI)  F for sequencing the pRCL  R for sequencing the pRCL |
| P41 | AGGGCAACCAGCATGAAGGC | F for Δ*gcvP* Cm cassette |
| P42 | GAAGCAGCTCCAGCCTACACTGGCTTAACGTCTGTGTCAT | R for Δ*gcvP* Cm cassette |
| P43 | ATGACACAGACGTTAAGCCAGTGTAGGCTGGAGCTGCTTC | F for Δ*gcvP* Cm cassette |
| P44 | TTACTGGTATTCGCTAATCGCATATGAATATCCTCCTTAG | R for Δ*gcvP* Cm cassette |
| P45 | CTAAGGAGGATATTCATATGCGATTAGCGAATACCAGTAA | F for Δ*gcvP* Cm cassette |
| P46 | GCCGCGTAATCAACATATT | R for Δ*gcvP* Cm cassette |
| P47 | TGAAGCCCTGGAAGTGCAGC | F for detection of Δ*gcvP* |
| P48 | CGACCTCTTCTGCCTGTCCA | R for detection of Δ*gcvP* |
| P49 | CTAGAATTCCGTTAAGCCGCAACTTATCC | F for complementation of Δ*gcvP* |
| P410 | TGCAAGCTTCTGGTATTCGCTAATCGGTA | R for complementation of Δ*gcvP* |
| P411 | ATGCCTGGCAGTTCCCTACT | F for sequencing the pBAD |
| P412 | TCTGACTTGAGCGTCGATTT | R for sequencing the pBAD |
| P51 | TAATTTCACCATGAAAAAGTTGTCAGCCCCGCTTATTCAATGAGGACAAGGTGTAGGCTGGAGCTGCTT | F for Δ*gcvT* Cm cassette |
| P52 | GCGCCCAAGCCACATGGCTTAACACCCGCTTCCACCAGCGCACGCCAGAACATATGAATATCCTCCTTAG | R for Δ*gcvT* Cm cassette |
| P53 | GGCAAAAGAGAACGATTGCG | F for detection of Δ*gcvT* |
| P54 | TCAACAAACACCATATCGCC | R for detection of Δ*gcvT* |
| P55 | CTAGAATTCCGTTAAGCCGCAACTTATCC | F for complementation of Δ*gcvT* |
| P56 | TGCAAGCTTTCACGCGACGGCTTTGCCGTT | R for complementation of Δ*gcvT* |
| P61 | AGCGGCGTTATCTGGCTCTGGAGAAAGCTTATAACAGAGGATAACCGCGCGTGTAGGCTGGAGCTGCTT | F for Δ*crp* Cm cassette |
| P62 | CGGGGGAAACAAAATGGCGCGCTACCAGGTAACGCGCCACTCCGACGGGACATATGAATATCCTCCTTAG | R for Δ*crp* Cm cassette |
| P63 | TGCATGTATGCAAAGGAC | F for detection of Δ*crp* |
| P64 | ATCAGTCTGCGCCACATCG | R for detection of Δ*crp* |
| P65 | CTAGAATTCACCGGAACCCACTGATGTAC | F for complementation of Δ*crp* |
| P66 | ACGTCTAGAACGAGTGCCGTAAACGACGA | R for complementation of Δ*crp* |
| P71 | AAAAAATCCCGCCGCTGGCGGGATTTTAAGCAAGTGCAATCTACAAAAGAGTGTAGGCTGGAGCTGCTT | F for Δ*hns* Cm cassette |
| P72 | TCTATTATTACCTCAACAAACCACCCCAATATAAGTTTGAGATTACTACACATATGAATATCCTCCTTAG | R for Δ*hns* Cm cassette |
| P73 | CACCCTTGGCACGGAATTTA | F for detection of Δ*hns* |
| P74 | GTGCTGCGAAATCATCGGTG | R for detection of Δ*hns* |
| P81 | ACTAAGCTTCGTTAAGCCGCAACTTATCC | F for -511 to 0 of *gcvTHP* cloning to pRCL(HindIII) |
| P82 | ACTGGATCCCTTGTCCTCATTGAATAAGC | R for -511 to 0 of *gcvTHP* cloning to pRCL(BamHI) |
| P91 | ACTAAGCTTAATCTGGATGGATGGGTCTG | F for -510 to 0 of *casA* cloning to pRCL(HindIII) |
| P92 | ACTGGATCCTTGTTCTCCTTCATATGCTC | R for -510 to 0 of *casA* cloning to pRCL(BamHI) |
| P101 | CTACTCGAGACCGGAACCCACTGATGTAC | F for -1270 to 0 of *crp* cloning to pRCL(HindIII) |
| P102 | ACTGGATCCGCGCGGTTATCCTCTGTTAT | R for -1270 to 0 of *crp* cloning to pRCL(BamHI) |
| P111 | AGCGAATTCGAACCTTTTAAATATATATG | F for expression of *cas3*(EcorI) |
| P112 | ACTCTCGAGTTTGGGATTTGCAGGGATGA | R for expression of *cas3*(XhoI) |
| P113 | TAATACGACTCACTATAGGG | F for sequencing the pET28a |
| P114 | GCTAGTTATTGCTCAGCGG | R for sequencing the pET28a |
| P121  P122 | CGCGGATCCATCCAGTGCGCCCGGTTTA  CCGGAATTCACATTAAGGTTGGTGGGTTGT | F for CRIPSR1 loci of MG1655 cloning to pGEX(BamHI)  R for CRIPSR1 loci of MG1655 cloning to pGEX(EcorI) |
| P131 | CGCGGATCCCTTGAGAAAGAGATAACGGG | F for CRIPSR2 loci of MG1655 cloning to pGEX(BamHI) |
| P132 | CCGGAATTCTGTGACTGGCTTAAAAAATC | R for CRIPSR2 loci of MG1655 cloning to pGEX(EcorI) |
| P141 | CGCGGATCCGGCGCGCCATGGAAACAAAGA | F for anti-vB_EcoS_SH2 spacer cloning to pGEX(BamHI) |
| P142 | CCGGAATTCTTAATTAAGGTACCGCGTCT | R for anti-vB_EcoS_SH2 spacer cloning to pGEX(EcorI) |
| P151 | GGGCTGGCAAGCCACGTTTGGTG | F for sequencing the pGEX |
| P152 | CCGGGAGCTGCATGTGTCAGAGG | R for sequencing the pGEX |
| P161 | AATAGCCCGCTGATATCATCGATAATACTAAAAAAACAGGGAGGCTATTAGTGTAGGCTGGAGCTGCTT | F for Δ*cas3* Cm cassette |
| P162 | TAAGTAGGGATAAACCGTTATTGGTCTTATTATCGTCATTGATAACAATCATTCCCGAAG CATATGAATATCCTCCTTAG | R for Δ*cas3* Cm cassette |
| P163 | TGTACATTGTGCACCTTCCC | F for detection of Δ*cas3* |
| P164 | TGAGGCTGTCTGGCGTTAAG | R for detection of Δ*cas3* |
| P171 | TGATGGTTTGGTCTCCTGTA | F for detection of CPR binding ability to *cas3* promoter |
| P172 | ATTTTAAAAATTATCTGTG | R for detection of CPR binding ability to *cas3* promoter |
| P173 | TAATAGCCTCCCTGTTTTTTTAGTATTATCGATGATATCAGCGGGCTATTTGAGTCTGCTGCCACAGAATATCGAATTACAGTGATA | R for mutation of core CPR binding area to *cas3* promoter |
| P181  P182 | ACTGGATCCATTAGCTAATTGTGCTGCGG  ACTAAGCTTGCGTTTGCAAATTGAGACT | F for overexpression of *leuO*(BamHI)  R for overexpression of *leuO*(HindIII) |

1. F = Forward Primer [↑](#footnote-ref-1)
2. R = Reverse Primer [↑](#footnote-ref-2)
