# Supplementary material for: Glycine Cleavage System and cAMP Receptor Protein Co-Regulate CRISPR/cas3 Expression to Resist Bacteriophage
Source: Viruses. 2020 Jan 13;12(1):90. doi: 10.3390/v12010090 (PMC7019758; doi:10.3390/v12010090)
Supplement: Supplementary file 1 [file viruses-12-00090-s001.zip › Supplementary Text S1.docx]

**SUPPLEMENTARY MATERIALS AND METHODS**

**Construction of *E. coli* MG1655Δ*lacZ*Δ*cas3*::*lacZ* reporter bacterium**

A MG1655Δ*lacZ* mutant was firstly constructed via λ Red system ([1](#_ENREF_1)). Briefly, a pair of primers containing 50 bp upstream and downstream regions of *lacZ* was used to amplify a chloramphenicol resistance cassette. The pKD46 plasmid was transferred into MG1655 followed by arabinose (Sigma) induction. The PCR products were transferred into induced MG1655 by electroporation and the target genes were subsequently replaced by the chloramphenicol resistance cassette. The chloramphenicol resistance cassette was finally removed by pCP20 plasmids. Next, a DNA fragment containing *lacZ* and chloramphenicol resistance cassette was amplified by overlapping PCR which was used to replace the open reading frame of *cas3*. The chloramphenicol resistance cassette was removed by pPC20 plasmids.

**Construction of Δ*gcvP*, Δ*gcvT*, Δ*crp*, Δ*hns*, Δ*cas3*, Δ*hns*Δ*gcvP*, Δ*hns*Δ*crp*, Δ*hns*Δ*cas3* and Δ*crp*Δ*gcvP* mutants via** **λ Red system and the complementation bacteria**

Mutants of Δ*gcvP*, Δ*gcvT*, Δ*crp*, Δ*hns*, Δ*cas3*, Δ*hns*Δ*gcvP*, Δ*hns*Δ*crp*, Δ*hns*Δ*cas3* and Δ*crp*Δ*gcvP* were constructed in a *E.coli* MG1655 via λ Red system. To construct complementation mutants, the open reading frame and assumed promoter of *gcvP*, *gcvT* and *crp* were cloned into modified pBAD-3×FLAG plasmid ([2](#_ENREF_2)). The complementary plasmids were transferred into their mutants. The mutants and their complementation mutants were identified by PCR.

**Preparation of antibodies**

The Cas3 was expressed in BL21 using pET28a vector and purified according to a previous report ([3](#_ENREF_3)). To obtain the polyclonal anti-Cas3, the rabbit was immunized with purified Cas3. After three immunizations, serum was collected and antibody titer was determined, which is used for Western blotting.

**References**

1. **Datsenko KA, Wanner BL.** 2000. One-step inactivation of chromosomal genes in Escherichia coli K-12 using PCR products. P Natl Acad Sci USA **97:**6640-6645.

2. **Wang SH, Yang DH, Wu XJ, Wang Y, Wang D, Tian MX, Li T, Qi JJ, Wang XL, Ding C, Yu SQ.** 2018. Autotransporter MisL of Salmonella enterica serotype Typhimurium facilitates bacterial aggregation and biofilm formation. Fems Microbiol Lett **365**.

3. **Wang ZF, Kong LC, Liu Y, Fu Q, Cui ZL, Wang J, Ma JJ, Wang HA, Yan YX, Sun JH.** 2018. A Phage Lysin Fused to a Cell-Penetrating Peptide Kills Intracellular Methicillin-Resistant Staphylococcus aureus in Keratinocytes and Has Potential as a Treatment for Skin Infections in Mice. Appl Environ Microb **84**.
