# Supplementary material for: Glycine Cleavage System and cAMP Receptor Protein Co-Regulate CRISPR/cas3 Expression to Resist Bacteriophage
Source: Viruses. 2020 Jan 13;12(1):90. doi: 10.3390/v12010090 (PMC7019758; doi:10.3390/v12010090)
Supplement: Supplementary file 1 [file viruses-12-00090-s001.zip › Fig. S1.docx]

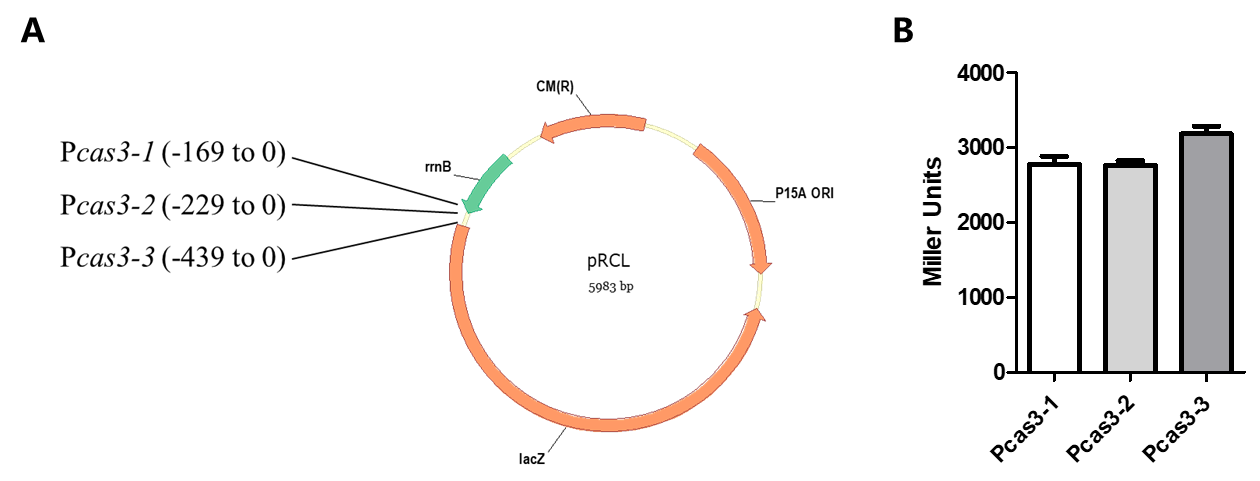


**Supplementary Figure 1.** Determination of *cas3* promoter active region. **(A)** Schematic diagram of reporter plasmids construction. **(B)** The activities of putative promoters of *cas3* were measured by β-galactosidase activity. The putative promoters with differences had similar activity. The shortest promoter was used in DNA pull-down assay. All the data were mean ± SEM of at least three replicates, and P value (*p*＜0.05) was analysed by t-test.
